# Effect of surgical pleth index-guided analgesia versus conventional analgesia techniques on fentanyl consumption under multimodal analgesia in laparoscopic cholecystectomy: a prospective, randomized and controlled study

**DOI:** 10.1186/s12871-021-01366-x

**Published:** 2021-06-04

**Authors:** Jian Guo, Weigang Zhu, Qinye Shi, Fangping Bao, Jianhong Xu

**Affiliations:** 1 Department of Anaesthesiology, The Fourth Affiliated Hospital, Zhejiang University, School of Medicine, 322000 Yiwu, Zhejiang China; 2 Clinical Laboratory, The Fourth Affiliated Hospital, Zhejiang University, School of Medicine, 322000 Yiwu, Zhejiang China

**Keywords:** Analgesia, Nociception, Fentanyl

## Abstract

**Background:**

The Surgical Pleth Index (SPI) is an objective tool that can reflect nociception-antinociception balance and guide the use of intraoperative analgesics. Multimodal analgesia has been neglected in many previous studies. The aim of this study was to compare fentanyl consumption using SPI-guided analgesia versus conventional analgesia techniques under multimodal analgesia in laparoscopic cholecystectomy.

**Methods:**

A total of 80 patients aged 18–65 years with American Society of Anaesthesiologists (ASA) grade I-II and a body mass index (BMI) of 18.5 to 30 kg/m2 who were scheduled for laparoscopic cholecystectomy under total intravenous anaesthesia from March 2020 to September 2020 were selected. Multimodal analgesia, including local infiltration of the surgical incision, nonsteroidal anti-inflammatory drugs and opioids, was adopted perioperatively. Fentanyl boluses of 1.0 µg/kg were administered to maintain the SPI value between 20 and 50 in the SPI group. By contrast, fentanyl boluses of 1.0 µg/kg were administered whenever the heart rate (HR) or mean arterial pressure (MAP) increased to 20 % above baseline or when the HR was greater than 90 beats per minute (bpm) in the control group. Preoperative and postoperative blood glucose, plasma cortisol and interleukin-6 (IL-6) levels were evaluated. Intraoperative haemodynamic events and propofol and fentanyl doses were noted. The extubation time, postoperative visual analogue scale (VAS) score, use of remedial analgesics and opioid-related adverse reactions were recorded.

**Results:**

In total, 18 of 80 patients withdrew for various reasons, and data from 62 patients were finally analysed. Intraoperative fentanyl consumption was significantly lower in the SPI group than in the control group (177.1 ± 65.9 vs. 213.5 ± 47.5, *P* = 0.016). The postoperative extubation time was shorter in the SPI group than in the control group (16.1 ± 5.2 vs. 22.1 ± 6.3, *P* < 0.001). Preoperative and postoperative blood glucose, plasma cortisol and IL-6 levels, intraoperative haemodynamic changes, postoperative VAS scores, remedial analgesic consumption and opioid-related adverse reactions were comparable in the two groups.

**Conclusions:**

Lower doses of fentanyl are required intraoperatively with shorter extubation times when SPI is used to guide intraoperative analgesia compared to conventional analgesia techniques under multimodal analgesia in laparoscopic cholecystectomy.

**Trial registration:**

Chictr.org.cn ChiCTR2000030145. Retrospectively Registered (Date of registration: February 24, 2020).

## Background

The perioperative trauma-related stress response is an unconscious response based on tissue damage. This response can be followed by changes in autonomic nerves as well as hormone and metabolic levels, and it can lead to a series of pathological and physiological changes in organs and systems [1, 2]. Traditionally, anaesthesiologists usually assess nociception by observing heart rate (HR), blood pressure, movement, muscle tension, etc., in patients, but there is still no effective measurement standard for the relationship between the above indicators and the level of nociception [3]. Park et al. [4] and Rogobete et al. [5] believed that when a patient’s HR or blood pressure exceeded 20 % of the baseline value or when the HR was greater than 90 bpm, nociception-antinociception was out of balance, so additional analgesic drugs were recommended. Empirical analgesia in clinical practice may have some disadvantages in patients’ rapid recovery, length of hospital stay, and financial costs [6]. The means of monitoring analgesia and nociception-antinociception balance are quite limited. In the past decade, perioperative monitoring of nociception has been the focus of researchers.

To date, several monitoring solutions have been commercialized, such as single-parameter scores (analgesia nociception index, skin conductance, pupillometry, nociceptive flexion reflex threshold), two-parameter scores (Surgical Pleth Index and qNOX) and multiparameter scores (nociception level index) [7, 8]. The surgical Pleth Index (SPI) has attracted considerable attention for its role in guiding the use of intraoperative opioids and predicting postoperative acute pain. SPI is an objective tool for monitoring nociception-antinociception balance during general anaesthesia. Under surgical stimulation, the changes in stroke volume and pulse rate increase, which are reflected in the monitoring waveform of pulse oxygen saturation. The change in crest amplitude is related to the change in stroke volume, while the change in the interpeak period is related to the change in pulse rate. Heart rate variability (HRV) and photoplethysmographic pulse wave amplitude (PPGA) are associated with monitoring nociception-antinociception balance and analgesia [2, 9]. The specific value of the SPI can be calculated by the following formula: SPI = 100 − 0.33×heart beat interval (HBI_norm_)-0.67×PPGA_norm_, in which HBI_norm_ and PPGA_norm_ are calculated from the peak interval and peak amplitude of pulse oxygen saturation monitoring, respectively, after conversion of the adaptive histogram [3]. The values of the SPI range from 0 to 100. During general anaesthesia, maintaining a value between 20 and 50 is generally recommended. When the SPI value is greater than 50 and exceeds 3–5 min, it routinely indicates that the noxious stimulation is too strong and that additional analgesic drugs are needed [10–14].

Studies have demonstrated that SPI can effectively monitor nociception-antinociception balance, guide the use of opioids, reduce intraoperative analgesic consumption, shorten the postoperative recovery time, and lower the incidence of opioid-related adverse reactions, which is conducive to the prognosis of patients [10–12, 15–17]. Furthermore, a few studies suggest that the SPI can reflect patients’ nociception-antinociception balance, but the actual clinical application value requires further study [13, 18, 19]. In many previous studies, multimodal analgesia has been neglected, and only one single opioid was always used to investigate the effects of SPI-guided analgesia. Therefore, the purpose of this study was to investigate the value of SPI-guided analgesia using fentanyl under multimodal analgesia in laparoscopic cholecystectomy with the aim of providing a basis for promoting the rapid recovery of patients undergoing day surgery.

## Methods

### Study design

This study was a prospective, single-centre, randomized, controlled, parallel-group clinical trial and was performed at the Fourth Affiliated Hospital Zhejiang University School of Medicine. The trial was approved by the Ethics Committee of the Fourth Affiliated Hospital Zhejiang University School of Medicine (approval number: K2020005). In addition, this trial was registered at the Chictr.org.cn registry system on 24 February 2020 (ChiCTR2000030145). The study adhered to the CONSORT guidelines, and informed written consent was obtained from all patients or their families.

### Participants

A total of 80 patients who were scheduled for laparoscopic cholecystectomy under total intravenous anaesthesia from March 2020 to September 2020 were selected. The patients were aged 18–65 years. No sex restrictions were employed. The patients were classified as American Society of Anaesthesiologists (ASA) grade I-II and had a body mass index (BMI) of 18.5–30. Exclusion criteria included patients with hypertension; a history of drug use that may affect autonomic nerve regulation, such as beta blockers or clonidine; severe cardiopulmonary diseases (pacemaker implantation, atrial fibrillation); endocrine diseases (diabetes mellitus, thyroid dysfunction); peripheral neuropathy or terminal coldness [20–22]; abnormal liver or kidney function; a history of opioid or other analgesic abuse; contraindications to parecoxib sodium use; changes in surgical procedures; required reoperations; and received atropine or other cardiovascular agents intraoperatively.

### Randomization

All patients were randomly divided into the SPI group and the control group using an Excel table, and the assignments were placed in a sealed envelope, which was done by a physician not involved in the study. The envelope was opened before anaesthetic induction by the investigators to determine specific grouping. Postanesthesia care unit (PACU) nurses were not aware of the test grouping to avoid affecting the authenticity of the test results.

### Anaesthesia, surgery and interventions

The baseline HR and mean arterial pressure (MAP) were defined as the average HR and MAP of the patients in the quiet state of the ward and were measured three times.

After the patient was admitted to the operating room, the peripheral veins were opened, and 5 mL of venous blood was extracted to detect preoperative blood glucose, plasma cortisol and IL-6 levels. Standard monitoring, including routine five-lead ECG, noninvasive blood pressure and pulse oximetry (Carescape TM monitor ® B650, GE Healthcare, Helsinki, Finland), was applied.

Total intravenous anaesthesia was induced with 0.05 mg/kg midazolam, 0.6 mg/kg rocuronium, 5 µg/kg fentanyl (under real body weight), and propofol via target controlled infusion (TCI) at a target plasma concentration of 4 µg/mL. A common tracheal tube was inserted (male 7.5-8.0#, female 7.0-7.5#) five minutes later. Mechanical ventilation was performed with a tidal volume of 8 mL/kg, an oxygen concentration of 60 %, a respiration rate (RR) of 12 times/min, and an adjusted RR to maintain a PaCO_2_ of 35–45 mmHg. The target concentration of propofol was adjusted initially to 2 µg/L after intubation. Propofol (TCI infusion), fentanyl (for specific use, see intervention measures), and rocuronium (0.3 mg/kg, given by anaesthesiologists as needed) were used for anaesthesia maintenance. The BIS value was maintained at 40–60 by adjusting the propofol TCI target concentration. Sugary liquids were forbidden intraoperatively to avoid compromising the results.

Haemodynamic events were defined as an increase in intraoperative HR or MAP to greater than 20 % of the baseline value. The number of intraoperative haemodynamic events was recorded in both groups. Additionally, 0.01 mg/kg atropine was used for severe bradycardia (HR < 45 bpm). A vasoactive agent (6 mg ephedrine or 40 µg phenylephrine) was used for hypotension (MAP < 50 mmHg) when unresponsive to fluid challenge. Moreover, these patients were eliminated from the analysis.

All operations were performed under a 3-hole laparoscope. The upper limit of laparoscopic pneumoperitoneum pressure was set at 12 cm H_2_O. At the end of the operation, propofol infusion was stopped, and 8 mg ondansetron was administered to prevent postoperative nausea and vomiting (PONV). Then, 5 mL venous blood was extracted to detect postoperative blood glucose, plasma cortisol and IL-6 levels. The patient was transferred to the PACU immediately after the operation. The endotracheal tube was removed when the patient was awake and able to follow the instructions. The breathing rate was greater than 10 times per minute, and the tidal volume was greater than 8 mL/kg. Once the patient had a visual analogue scale (VAS) score ≥ 4 points in the PACU, 1.0 µg/kg fentanyl under the real body weight was injected for remedial analgesia. The extubation time, postoperative VAS scores and remedial analgesics and opioid-related adverse reactions were recorded by the PACU nurses.

Multimodal analgesia was adopted perioperatively. Each endoscopic incision was anaesthetized with 0.75 % ropivacaine (3 mL) for local infiltration before the surgery. Parecoxib sodium (40 mg) was injected intravenously for advanced analgesia one minute after intubation.

In the SPI group, when the SPI value was greater than 50 for the first time and when the duration was greater than 3 min, 1.0 µg/kg fentanyl under the real body weight was added. When the SPI value was greater than 50 again and when the duration was more than 5 min, 1.0 µg/kg fentanyl was added once again, and this was repeated until the SPI value was between 20 and 50 [13, 14].

In the control group, when the MAP increased to greater than 20 % of the baseline value, the HR increased to greater than 20 % of the baseline value, an HR of greater than 90 bpm appeared for the first time or the duration was more than 3 min, and 1.0 µg/kg fentanyl under the real body weight was added. When the above situation appeared again and the duration was greater than 5 min, 1.0 µg/kg fentanyl was added once again. The loop continued until the increase in MAP was not greater than 20 % of the baseline value, the increase in HR was no greater than 20 % of the baseline value or the HR was less than 90 bpm [4, 5].

### Outcomes

The primary outcomes were the differences in intraoperative fentanyl consumption and postoperative extubation time between the SPI group and the control group. Secondary outcomes included differences in terms of haemodynamics (including MAP and HR one minute after intubation, one minute after incision, one minute after artificial pneumoperitoneum, and one minute after abdominal venting, as well as haemodynamic events), postoperative visual analogue scale (VAS) score, use of remedial analgesics, opioid-related adverse reactions and postoperative blood glucose, plasma cortisol and interleukin-6 (IL-6) levels in the two groups.

### Sample size estimation and Statistical methods

The primary endpoint in this study was intraoperative fentanyl consumption. The sample size calculation was based on the results of a preliminary experiment with five cases in each group. In the preliminary experiment, intraoperative fentanyl consumption (mean ± standard deviation) was 2.0 ± 0.7 µg/kg in the SPI group and 2.6 ± 0.89 µg/kg in the control group. Therefore, the effect size of two groups was 0.74. The required minimum sample size for each group was 30 (calculated by t test, 2-sided test, a level of significance of 0.05, and a power of 0.8). The total sample size was 80 to take care of approximately 30 % dropouts.

Statistical testing was conducted with SPSS 19.0. Categorical variables are presented as absolute numbers and percentages. The Kolmogorov-Smirnov test was used to detect whether the data conformed to a normal distribution. Normally distributed data are expressed as the mean ± standard deviation $\left(\overline{x}\pm \mathrm{sd}\right)$, and nonnormally distributed data are expressed as medians [interquartile ranges]. Normally distributed data were compared among multiple groups using single-factor analysis of variance (ANOVA). Nonnormally distributed data were compared among multiple groups using the Kolmogorov-Smirnov test. Grade count data were tested by χ2. A difference of *P* < 0.05 was considered statistically significant.

## Results

### Participants

**Fig. 1:**
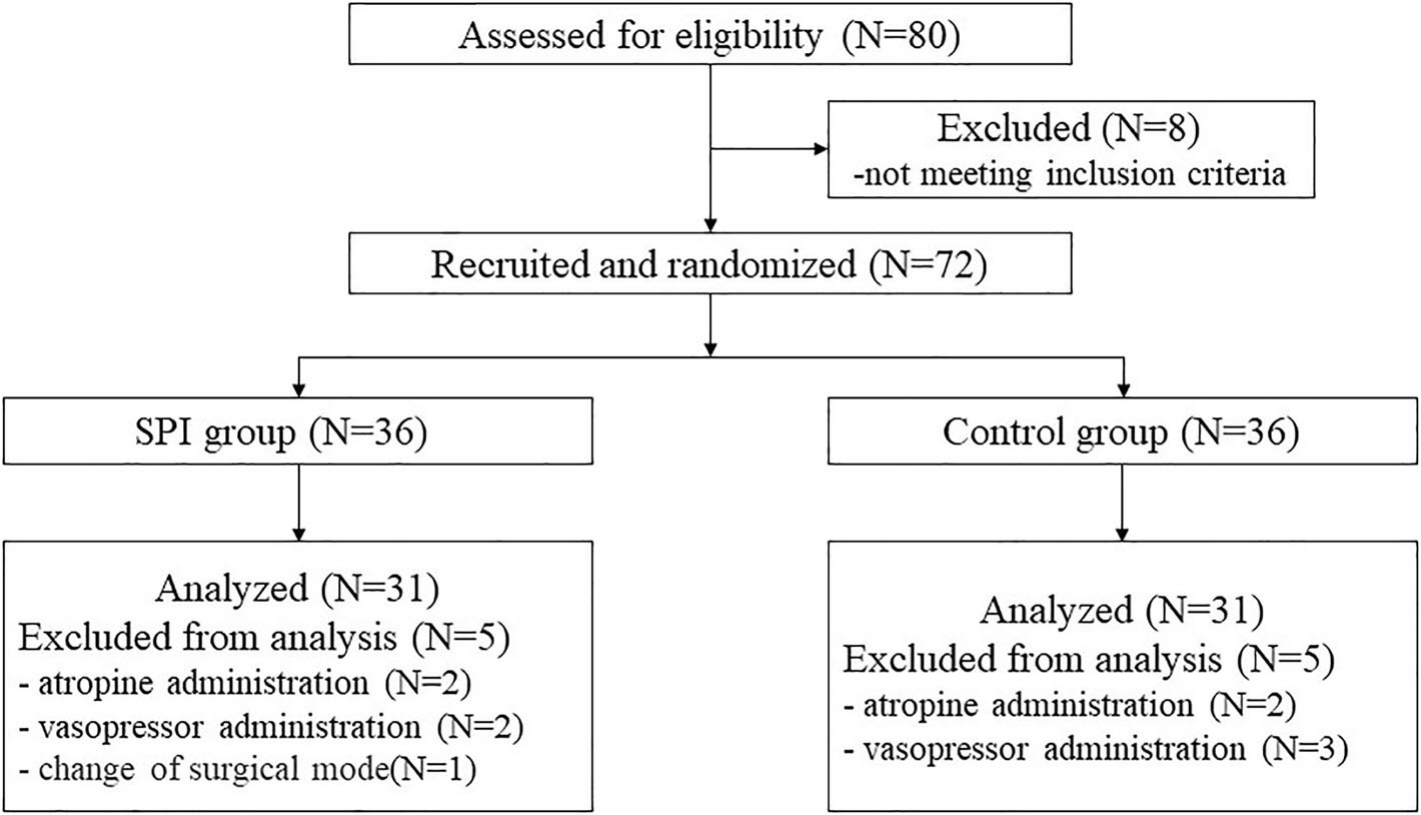
CONSORT flow diagram of participants allocation. In total, 18 of 80 patients withdrew for various reasons, and 31 patients from the SPI group and 31 patients from the control group were eventually included in final analysis

A total of 80 patients were selected from March 2020 to September 2020. In total, 18 of these patients withdrew for various reasons, and data from 62 patients were finally analysed, which is shown in the CONSORT flow diagram (see Fig. 1).

### Basic information

The basic information of the patients is summarized in Table 1. No significant differences in age, sex, height, weight, BMI, or ASA classification were noted between the SPI group and the control group (*P* > 0.05) (see Table 1).

**Table 1:** Characteristics of the enrolled patients (*N* = 62)

	SPI group (*N* = 31)	Control group (*N* = 31)	*P*-value
Age (years)	47.1 ± 11.6	48.8 ± 13.4	0.616
Sex (male/female)	16/15	11/20	0.153
Height (cm)	163.0 ± 9.2	164.1 ± 7.2	0.606
Weight (kg)	63.4 ± 11.7	63.2 ± 12.0	0.959
BMI (kg/m^2^)	23.7 ± 2.8	23.3 ± 3.6	0.657
ASA classification (grade I/II)	15/16	16/15	0.500

Data are expressed as the frequencies or means ± SDs, as appropriate

Abbreviations: *BMI *body mass index; *ASA *American Society of Anaesthesiologists

### Surgery and anaesthesia information

Information concerning surgery and anaesthesia is listed in Table 2. No significant differences in the duration of surgery or anaesthesia, bleeding volume, infusion volume, or intraoperative doses of propofol were noted between the two groups (*P* > 0.05) (see Table 2). Intraoperative fentanyl consumption was significantly reduced in the SPI group compared with the control group (177.1 ± 65.9 vs. 213.5 ± 47.5, *P* = 0.016) (see Table 2).

**Table 2:** Information on surgery and anaesthesia (*N* = 62)

	SPI group (*N* = 31)	Control group (*N* = 31)	*P*-value
Duration of surgery (min)	45.1 ± 14.0	45.5 ± 16.9	0.935
Duration of anaesthesia (min)	67.3 ± 18.4	66.8 ± 18.4	0.913
Bleeding volume (ml)	163.0 ± 9.2	164.1 ± 7.2	0.606
Infusion volume (ml)	696.7 ± 236.6	741.9 ± 192.3	0.413
Propofol consumption (mg)	410.0 ± 105.0	452.0 ± 121.2	0.150
Intraoperative fentanyl consumption (µg)	177.1 ± 65.9	213.5 ± 47.5	0.016*

* The difference in the total fentanyl consumption and intraoperative fentanyl consumption between the two groups was significant, *P* < 0.05

### Perioperative Haemodynamics

The perioperative haemodynamic correlations are shown in Table 3. No significant differences in terms of MAP or HR preoperation, one minute after intubation, one minute after incision, one minute after artificial pneumoperitoneum, or one minute after abdominal venting were noted between the two groups (*P* > 0.05) (see Table 3). Haemodynamic events were also comparable between the two groups (*P* > 0.05) (see Table 3).

**Table 3:** Comparison of haemodynamics in the enrolled patients (*N* = 62)

	SPI group (*N* = 31)	Control group (*N* = 31)	*P-*value
Preoperation			
Baseline MAP (mmHg)	84.9 ± 7.5	85.7 ± 6.5	0.658
Baseline HR (bpm)	73.6 ± 8.0	73.7 ± 6.3	0.931
One minute after intubation			
MAP (mmHg)	84.9 ± 12.9	88.0 ± 15.0	0.384
HR (bpm)	74.1 ± 8.3	74.5 ± 10.3	0.872
One minute after incision			
MAP (mmHg)	90.2 ± 9.7	91.9 ± 9.9	0.490
HR (bpm)	69.2 ± 9.1	70.4 ± 7.6	0.579
One minute after artificial pneumoperitoneum			
MAP (mmHg)	105.8 ± 13.9	106.5 ± 13.0	0.851
HR (bpm)	78.6 ± 9.6	78.6 ± 10.6	0.980
One minute after abdominal venting			
MAP (mmHg)	86.9 ± 10.5	88.9 ± 10.2	0.449
HR (bpm)	68.5 ± 9.8	68.4 ± 8.7	0.978
Haemodynamic events			
Tachycardia events (N.) (0/1/2/3/4/5) ^a^	12/7/2/4/5/1	13/5/1/5/6/1	0.841
Hypertension events (N.) (0/1/2/3/4/5) ^a^	8/4/4/8/5/2	10/3/3/7/6/2	0.591

Data are expressed as the frequencies or means ± SDs, as appropriate. Haemodynamic events were defined as an intraoperative increase in HR or MAP greater than 20 % of the baseline value

^a^ Patients with different times of tachycardia events or hypertension events were counted in both groups

### Postoperative pain assessment, use of remedial analgesics, opioid-related adverse reactions

Postoperative information of the patients is shown in Table 4. The postoperative extubation time was shorter in the SPI group than in the control group (16.1 ± 5.2 vs. 22.1 ± 6.3, *P* < 0.001) (see Table 4). No hypersedation, respiratory depression or itching occurred in any patients within the PACU. No significant differences in PONV or postoperative VAS scores were noted between the two groups (*P* > 0.05) (see Table 4). Only three patients in the SPI group and two patients in the control group received a single dose of fentanyl (1.0 µg/kg) for remedial analgesia. No significant differences in remedial analgesic administered were noted between the two groups (*P* > 0.05) (see Table 4).

**Table 4:** Postoperative parameters of the enrolled patients (*N* = 62)

	SPI group (*N *= 31)	Control group (*N *= 31)	*P*-value
Extubation time (min)	16.1 ± 5.2	22.1 ± 6.3	<0.001*
PONV (N.)	2	3	0.500
VAS score			
Resting VAS score	1.9 ± 1.2	1.8 ± 1.2	0.838
Active VAS score	3.8 ± 1.0	3.7 ± 1.0	0.811
Remedial analgesics (N.)	3	2	0.500

Data are expressed as the frequencies or means ± SDs, as appropriate

* The difference in extubation time between the two groups was significant, *P* < 0.001

### Levels of nociception stimulation-related factors before and after surgery

The comparison of blood glucose, plasma cortisol and IL-6 levels at different time points between the two groups is shown in Table 5. No significant differences in terms of preoperative or postoperative blood glucose, plasma cortisol or IL-6 levels were noted between the two groups (*P* > 0.05) (see Table 5).

**Table 5:** Comparison of blood glucose, plasma cortisol and IL-6 levels in the enrolled patients (*N* = 62)

	SPI group (*N *= 31)	Control group (*N* = 31)	*P*-value
Preoperation			
Blood glucose (mmol/L)	5.3 ± 0.5	5.5 ± 0.7	0.308
Plasma cortisol (nmol/L)	98.5 ± 36.5	92.4 ± 41.0	0.537
IL-6 (pg/ml)	2.1 [1.1, 3.2]	2.1 [1.2, 5.1]	0.851
Postoperation			
Blood glucose (mmol/L)	6.3 ± 0.8	6.3 ± 0.8	0.988
Plasma cortisol (nmol/L)	127.8 ± 40.5	137.7 ± 51.9	0.404
IL-6 (pg/ml)	6.5 [3.0, 10.2]	7.8 [3.3, 18.6]	0.253**#**

Normally distributed data are expressed as the means ± SDs, and nonnormally distributed data are expressed as medians [interquartile ranges], as appropriate

Abbreviation: *IL-6 *Interleukin-6

**#** The Kolmogorov-Smirnov test was used to detect whether a difference existed between the two groups

## Discussion

The aim of this study was to compare fentanyl consumption using SPI-guided analgesia versus conventional analgesia techniques under multimodal analgesia in laparoscopic cholecystectomy. In our study, we found that lower doses of fentanyl were required intraoperatively with shorter extubation times when SPI was used to guide intraoperative analgesia compared to conventional analgesia techniques under multimodal analgesia.

This study confirmed that SPI could reduce the intraoperative opioid dosage (177.1 ± 65.9 vs. 213.5 ± 47.5, *P* = 0.016) and shorten the postoperative extubation time of patients (16.1 ± 5.2 vs. 22.1 ± 6.3, *P* < 0.001) compared with conventional analgesia techniques under multimodal analgesia in laparoscopic cholecystectomy, which was consistent with many previous studies [10–12, 15–17]. However, compared with the previous study of SPI-guided analgesia in laparoscopic cholecystectomy, we found lower intraoperative opioid consumption, shorter recovery time, and lower remedial analgesic consumption in the SPI group in our study [14]. The main reasons for this phenomenon may be as follows. First, and most importantly, compared to the previous study using a single opioid, multimodal analgesia was adopted in our study, which was widely used in clinical situations and recommended by guidelines. Due to the application of multimodal analgesia, the use of remedial analgesics was relatively low in our study. Second, instead of 2 µg/kg fentanyl, a greater dose of 5 µg/kg fentanyl was chosen as the induction dose. Orotracheal intubation is one of the most severe noxious stimuli evoked during general anaesthesia, which is associated with serious cardiovascular and cerebral side effects and can be managed most effectively by providing sufficient analgesia [23, 24]. In our preliminary experiment, we chose a dose of 2 µg/kg fentanyl as the induction dose, which led to severe cardiovascular reaction (HR or MAP was even greater than 50 % of the baseline value in some patients). We believed that 2 µg/kg fentanyl for anaesthesia induction was significantly insufficient. Therefore, 5 µg/kg fentanyl was chosen as the induction dose based on our experience. However, 8 patients (13 %) still had a significant intubation reaction (HR or MAP exceeding 20 % of baseline values one minute after endotracheal intubation). The difference in the induction dose of fentanyl may be related to population differences [25]. Third, the additional dose of fentanyl in our study was greater than that in the previous study. Through the preliminary experimental study, we found that the additional dose of 0.5 µg/kg fentanyl used in the previous study could not satisfy the patients’ requirements, which caused multiple additions. Thus, we selected 1.0 µg/kg fentanyl as an additional dose to reduce the incidence of haemodynamic events and excessive multiple additions. Fourth, the amount of propofol was not included as a relevant influencing factor in the previous study. Propofol can affect patients’ resuscitation, which may have an influence on the results.

In our study, the haemodynamic indexes of our patients increased one minute after intubation, incision and artificial pneumoperitoneum, among which the increase after artificial pneumoperitoneum was the most obvious, which was consistent with nociception stimulation during the operation. In both groups, haemodynamic changes were comparable at important event nodes. No significant difference in the incidence of haemodynamic events was noted between the two groups. In addition, we also found that haemodynamic changes in most patients were synchronized with SPI changes during important procedures. However, the SPI values did not change significantly in a small number of patients with haemodynamic changes, which may be related to the difference in opioid consumption. Meng Wang et al. [26] found that SPI values were elevated under noxious stimulation by intubation and incision, but this change was not predictive of the haemodynamic responses to intubation or incision, which was similar to our study.

The stress response caused by surgical traumatic nociception stimulation is related to the activation of the sympathetic adrenal medulla and hypothalamic-pituitary-adrenal axis and leads to changes in the balance of inflammatory and anti-inflammatory factors, which can be clinically reflected by measuring nociception stimulation-related inflammatory factors or cytokines, such as blood glucose, cortisol, tumour necrosis factor, and interleukins [27–31]. Theoretically, the above factors could be measured to reflect the patient’s state of nociception stimulation and guide the use of analgesic drugs. However, the measurement of these factors is relatively late and cannot timely reflect the patient’s analgesic stress state. In our study, no differences were found in blood glucose, plasma cortisol or IL-6 levels between the two groups, which may be related to the reduced duration of laparoscopic cholecystectomy, the lower levels of trauma and stress, and the evaluation time. Furthermore, the anaesthesia induction dose of fentanyl in our study was relatively large, and the use of a large dose of opioids might inhibit the stress level of surgery-related nociception stimulation, leading to the delay of the secretion of related inflammatory factors or hormones.

Perioperative multimodal analgesia uses combinations of analgesic medications that act on different sites and pathways in an additive or synergistic manner to achieve pain relief to promote the rapid recovery of patients [32]. Compared to the previous study (58 of 133 patients received remedial analgesics), only 5 of 62 patients used remedial analgesics, which might be related to multimodal analgesia [14]. In addition, some of the latest research confirms that an SPI value of approximately 30 exhibits the best sensitivity/specificity for predicting moderate-to-severe pain in the PACU [33, 34]. However, we did not find a similar phenomenon, which might also be related to the application of multimodal analgesia. Reducing intraoperative stress caused by nociceptive stimuli is beneficial to shorten the hospital stay of patients and promote rapid recovery [35]. Combined with the relatively stable haemodynamic fluctuations, lower postoperative VAS score, less remedial analgesic consumption, few opioid-related adverse reactions and minimal changes in blood glucose, cortisol and interleukin, we can infer that the stress level of the patients was well controlled in our study, indicating that perioperative multimodal analgesia and SPI-guided analgesia we chose were beneficial to the patients.

Many factors can interfere with the accuracy of the value of the SPI. Unlike in adults, given the high excitability of the central nervous system, intraoperative SPI values are often recommended to maintain a lower level of relevance in children [4, 36]. In addition, the surgical position and the use of some vasoactive drugs (nicardipine) during surgery may also affect the accuracy of the SPI value, confusing the judgement of patients’ nociception-antinociception balance [37, 38]. In our study, intraoperative use of atropine and cardiovascular agents also affected the HR or MAP of patients, which may interfere with the accuracy of SPI and lead to differences in the use of analgesics. Therefore, attention should be given to relevant interference factors when SPI is used to guide intraoperative analgesia.

Our study has some limitations. First, only some stress-related factors were evaluated in our study. Second, the time node of this study was the patient’s emergence from the PACU, and the changes in postoperative-related inflammatory factors were not evaluated. Third, the use of anti-inflammatory drugs, such as hormones, before surgery was not included in our study, which potentially influenced the study results. Fourth, a relatively large dose of fentanyl was used for anaesthesia induction, which may lead to a decrease in the intraoperative demand for opioids and postoperative remedial analgesics, resulting in a bias in the study results. Fifth, this study was a single-centre, randomized, prospective study; thus, the results need to be further confirmed by large-sample, multicentre studies. In our study, we found that the SPI value increased more during traction and resection of the gallbladder than during surgical excision. The SPI may be more sensitive to visceral pain, which represents a future research direction.

## Conclusions

In summary, lower doses of fentanyl usage are required intraoperatively with shorter extubation times when SPI is used to guide intraoperative analgesia compared to conventional analgesia techniques under multimodal analgesia in laparoscopic cholecystectomy. SPI can provide assistance for intraoperative monitoring of nociception-antinociception balance, guiding the use of opioids and promoting the rapid recovery of patients.

## Data Availability

All the data used and analyzed are available from corresponding authors upon the reasonable request.
